# Evaluation of coronal shift as an indicator of neuroaxial abnormalities in adolescent idiopathic scoliosis: a prospective study

**DOI:** 10.1186/1748-7161-9-9

**Published:** 2014-07-19

**Authors:** Mohsen Karami, Soodeh Sagheb, Keyvan Mazda

**Affiliations:** 1 Talegani Hospital, Shahid Beheshti University of Medical Sciences, Tehran, Iran; 2 Imam Hosein Hospital, Shahid Beheshti University of Medical Sciences, Tehran, Iran; 3 Robert Debre Hospital, Paris 7 University, Paris, France

**Keywords:** Scoliosis, Chiari malformation, Magnetic resonance imaging, Syringomyelia

## Abstract

**Background:**

In previous studies, many indicator factors have been proposed to select patients who need an MRI screening of the spinal canal. In current study, the clinical and radiologic factors including coronal parameters of the curve were evaluated to find out which indicator is more important.

**Methods:**

A prospective study included 143 consecutive patients with the diagnosis of adolescent idiopathic scoliosis who were treated between 2010 and 2013 at our spinal clinics. Only patients with normal or subtle neurologic findings were included. All patients were evaluated by a total spine MRI protocol for examination of neuroaxial abnormalities. Known indicators and also coronal shift were analysed in all patients with or without abnormal MRI.

**Results:**

The incidence of neuroaxial abnormalities was 11.9% (17 of 143); only 5 patients (3.5%) were operated to treat their neuroaxial problem. The significant indicators of the abnormalities in our patients were: younger age at onset, asymmetric superficial abdominal reflex and, coronal shift more than 15 mm (*P = 0.03*). Some previously known indicators like atypical curves, male gender, double curves and absence of thoracic lordosis were not different between two groups of the patients.

**Conclusions:**

A total spine MRI is recommended at presentation in patients with younger age, abnormal neurologic findings and severe coronal shift.

## Background

Although the etiology of "idiopathic" scoliosis is still uncertain, the association between scoliosis and neuroaxial abnormalities such as syringomyelia or Chiari malformations has been well established. With the development of Magnetic Resonance Imaging (MRI), neuroaxial abnormalities are increasingly being found in patients with idiopathic scoliosis. Many associated clinical signs and radiologic parameters have been considered as indicators of neuroaxial abnormalities in these patients. Because of the uncertainty of the correlation among these parameters and the existing of neuroaxial anomalies, many surgeons prefer to perform MRI study on all idiopathic scoliosis patients.

Although all of the detected abnormalities may not require active intervention, preoperative detection is important in patients who are undergoing manipulative correction [1-4]. The most sensitive way to investigate neuroaxial abnormalities is MRI. But its routine use remains controversial, and current indications to perform MRI in "idiopathic" scoliosis vary from center to center.

The goals of this study were to (a) document the incidence of neuroaxial abnormalities in adolescent idiopathic scoliosis and (b) study the association of neuroaxial abnormalities with the curve characteristics such as coronal shift.

## Materials and methods

We prospectively studied 143 patients who were diagnosed as Adolescent Idiopathic Scoliosis (AIS) and were admitted at our university hospitals between 2010–2013. AIS was considered when the beginning of the disease was between age of 10 and 16. These patients were selected after excluding those with failure of formation or segmentation of the spine on plain radiographs (congenital scoliosis), those with associated conditions or syndromes and those who have had neurologic findings on their clinical examination other than Asymmetric Superficial Abdominal Reflex (ASAR).

Every scoliosis patient has been studied at our institutions based on our established “spine protocol”. It includes a throughout history taking and physical examination, standing potero-anterior and lateral radiographs as well as bending and tractional films and laboratory tests including coagulation tests. The x-rays have been taken using SRS recommendations [5]. Total spine MRI is also included in the protocol for every patient. The radiographs have been studied by the SpineView software to measure coronal and sagittal curves angles. Coronal shift, Lenke [6] and King classification [7] were also determined by the software.

Coronal plane curve patterns were classified as high thoracic, thoracic, thoracolumbar or lumbar curves on the basis of the location of the apexes. Each existing curve has been marked by one operator and the measurements were done by the software.

Total spine MRI was obtained and was interpreted by an experienced radiologist for any abnormalities in the brainstem and spinal cord. Arrnold chiari-I malformation was considered when the tonsillar herniation was 5 mm or more below the level of foramen magnum. Location and dimensions of the syringomyelia was also noted and the level of conus medullaris was documented. In doubtful cases, MRI with Gadolinium was also obtained to better define existing abnormalities. The presence and type of anomaly in the MRI were correlated to the age at presentation, curve characteristics (sagittal and coronal profiles), typing of scoliosis and so on.

### Statistical analysis

Two different groups of patient were defined. First group, in whom MR imaging revealed neuroaxial abnormalities and the second group, in whom total spine MRI was normal. All variables were compared in these two groups using one way ANOVA and the Fisher exact test. The differences with a *P* value of <0.05 were considered as statistically significant.

## Results

Our patients consist of 143 patients who were diagnosed as having AIS at their presentation. The patients were classified according to Lenke and King classification. The most frequent type according to Lenke classification was type one in 70 patients (49%) followed by type two in 44 patients (30.7%) and then type 5 in 16 patients(11.2%), type 4 and 6 each one in 5 patients (3.5%) and the least common type was type 3 in 3 patients (2%). According to King classification, there were 55 patients (38.4%) with type 2 which was the most frequent one and then was type 3 and 4 each one in 25 patients (17.5%), 18 patients were type 5 (12.5%), 15 patients (10.5%) were unclassified and the least common type was type 1 in 5 patients (3.5%). There were no significant difference among King and Lenke types and occurrence of neuroaxial abnormalities.

The incidence of neuroaxial abnormalities was 11.9% (17/143). The abnormalities related to disc or irregularity of the end plates (6 patients, 4%) were not considered as neuroaxial abnormality. The most common neuroaxial abnormality was syringomyelia in thirteen patients (9.7%), in which two were associated with Arnold-Chiari malformation type I (Table 1). The most frequent region of isolated syringomyelia was thoracic region (8 patients, 5.5%).

**Table 1:** Detected neuroaxial abnormalities in patients with AIS

	**Frequency**	**Percent**
**Thoracic syringomyelia**	8	5.5
**Cervical syringomyelia**	2	1.4
**Arnold Chiari type I + cervical s syringomyelia**	2	1.4
**Isolated Arnold Chiari type I**	1	0.7
**Mild tonsillar herniation**	2	1.4
**Tetherd cord**	1	0.7
**Total**	17	11.9

The mean age at presentation was 13.9 (10.5-17) years in patients with neuroaxial abnormalities as compared to 14.9 (10.7-19) years in those who did not have any abnormalities (*P = *0.05).

Three curves were determined and measured as described by Lenke classification. The mean of the proximal thoracic curve was 28.2° (range 8° to 67°) in patients without neuroaxial problem and 19.6° (range 17° to 21°) in those who have had abnormalities. The middle thoracic curve was measured as 55.7° (range 15° to 128°) in patients without neuroaxial abnormalities as compared to 58.5° (range 40° to 85°) in those patients who have had abnormalities. These proximal and mid thoracic curves did not have any correlation with neuroaxial abnormalities. On contrary, lumbar or thoracolumbar curve mean was 39.6° (range 13° to 75°) in patients without abnormalities which was significantly less than those patient who had neuroaxial abnormalities with the mean of 53.5° (range 39° to 67°) (*P value* = 0.03) (Table 2). The direction of the curves was not associated with the occurrence of neuroaxial abnormalities.

**Table 2:** Descriptive statistics in patients with or without abnormal MRI findings

MRI*		**Minimum**	**Maximum**	**Mean**	**Std. deviation**
-	**Thoracic sagittal angle**	2	62	26.21	14.206
	**Lumbar sagittal angle**	13	67	45.02	12.732
	**Age of patients**	10	19	14.91	1.943
	**Main thoracic curve angle**	15	128	55.74	18.598
	**Coronal shift**	0	56	18.56	13.810
	**Proximal thoracic curve**	8	67	28.29	12.701
	**Distal L/TL curve**	13	75	39.66	12.656
+	**Thoracic sagittal angle**	11	23	16.67	6.028
	**Lumbar sagittal angle**	16	47	35.67	17.098
	**Age of patients**	10	17	13.95	1.900
	**Main thoracic curve angle**	40	85	58.57	16.440
	**Coronal shift**	34	41	37.67	3.512
	**Proximal thoracic curve**	17	21	19.67	2.309
	**Distal L/TL curve**	39	67	53.50	14.059

*MRI - : Negative neuroaxial abnormalities, +: Positive neuroaxial abnormalities.

Sagittal profile of the spine was also evaluated in this study. Thoracic sagittal angles (T1-T12) as well as lumbar sagittal angle were measured in both groups of patients. The mean of thoracic sagittal angle was 26.2° (range 2° to 62°) in all patients without neuroaxial abnormalities as compared to 16.7° (range 11° to 23°) in those patients who had abnormalities. This difference was not statically significant (P *= 0.2*).

**Figure 1:**
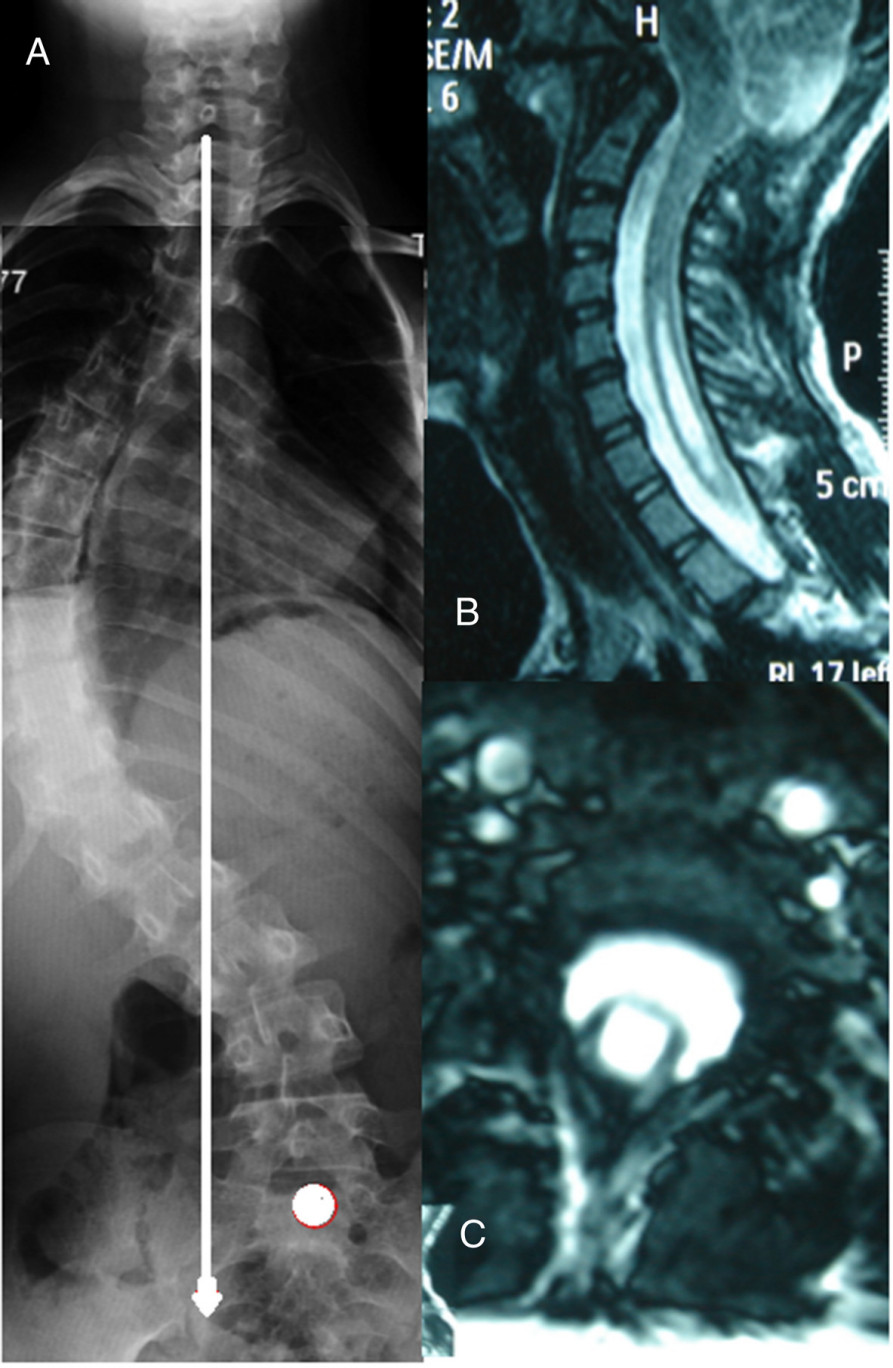
**Twelve-year-old female patient with adolescent idiopathic scoliosis who underwent decompressive surgery for her neuroaxial abnormalities. A**, Preoperative radiograph showed significant coronal imbalance. **B**, **C**, Preoperative MRI sections showing Arnold-Chiari Type I malformation and cervical syringomyelia.

The major point in this study was the correlation between coronal imbalance and neuroaxial abnormalities.The mean of radiologic transverse coronal shift from plumb line was 18.5 mm (range 0 to 56 mm) in patients without neuroaxial abnormalities which was significantly less than those who have had neuroaxial abnormalities with the mean of 37.6 mm (range 34 to 41 mm) (P value = 0.02) (Table 2) (Figure 1). Significant radiological coronal imbalance was defined a 15 mm shift of C7 plumb line from Center Sacral Vertical Line (CSVL) by authors. There were 46 patients (32%) who have had coronal imbalance. Thirteen patients among 17 patients who have had neuroaxial abnormalities, have had coronal shift of more than 15 mm that means two third of patients with abnormal MRI findings have had coronal imbalance (*P = 0.03*) odds ratio of 6.4, 95% confidence interval 1.34–30.37.

On physical examination 6 patients (4.1%), have had an Asymmetric Superficial Abdominal Reflex (ASAR). All of them had abnormal neuroaxial findings on MR imaging. Two patients who had Arnold Chiari malformation and cervical syringomyelia were operated for foramen magnum decompression before scoliosis correction. Their syringomyelia was disappeared after decompressive surgery. Patients with isolated syringomyelia (eight patients) or mild tonsillar herniation (less than 5 mm) (two patients) with normal neurologic examination, underwent only a scoliosis surgery. None of them had neurologic complication during or after the surgery. Three patients, who had isolated syringomyelia with abnormal neurologic sign, underwent decompressive surgery by our neurosurgeon. Therefore only 5 patients (3.5%) have been treated neurosurgically before the scoliosis surgery, all of them have had ASAR.

## Discussion

The current guidelines for MRI screening in idiopathic scoliosis are not well defined, the recommended indications for performing MRI in the literature include early onset [8,9] atypical curvature [10-14], double thoracic curve (King type-5) [15,16], rapid progression [17], male gender [18], the absence of thoracic apical segment lordosis [19,20] and abnormal neurologic findings [21,22]. However, the whole clinico-radiologic parameters of idiopathic scoliosis have not been studied yet.

We collected previous valuable studies about the indicators of the neuroaxial abnormalities in idiopathic scoliosis. The studies with more than 26 cases were summarized in Table 3. These studies are mostly retrospective or prospective case series. The incidence of neuroaxial abnormalities was reported between 1.8 to 31% which depends on the inclusion criteria among various studies.

**Table 3:** Short description of previous studies

**Study**	**Type of study**	**Study group**	**No of patient**	**Neurologic exams**	**MRI protocol**	**Neuroaxial problem%**	**Age**	**Sex**	**Atypical curves**	**curve magnitude**	**Double curve**	**Neurologic findings**	**Pain**	**Thoracic kyphosis**
Geissele et al.	Prospective	AIS*	27	NR†	Brain stem	27%	NR	NR	NR	NSD‡	NR	NR	NR	NR
Spine 1991 [23]														
Evans et al. JBJS-B 1996 [8]	Prospective	JIS + AIS*	31	Nystagmus , Ataxia, ASAR**	Total spine	31%	NSD	NSD	SD¥, left curves	NR	NR	NSD	NR	NR
Mejia et al. JPO 1996 [24]	Prospective	JIS + AIS	29	Only NL exams	Total spine	7%	NR	NR	Only left curves	NR	NR	NR	NR	NR
Shen et al.	Prospective	AIS	72	Only NL exams	Total spine	4%	NR	NR	NR	NR	NR	NR	NR	NR
JPO 1996 [25]														
Maiocco et al. Spine 1997 [16]	Prospective	AIS	45	Only NL exams	Total spine	4%	NR	NR	NR	NR	NR	NR	NR	NR
Winter et al. Spine 1997 [10]	Prospective double blind	AIS	140	Only NL exams	Total spine	2.8%	NR	NR	Rt curves included	NR	NR	NR	NR	NR
Gupta et al. Spine 1998 [22]	Prospective + retrospective	IIS* + JIS	98	Only NL exams	Total spine	18-20%	NR	NR	NR	NR	NR	NR	NR	NR
Dobbs et al. JBJS-A 2002 [26]	Retrospective	IIS	46	Only NL exams	Total spine	21.7%	NSD	NSD	NSD	NSD	NR	NR	NR	NR
Ouellet et al. Spine 2003 [27]	Retrospective	JIS + AIS	93	NR	Total spine	NR	NSD	SD, male	SD, left curves	NR	NR	NR	NR	SD
Do et al. JBJS-A 2003 [28]	Prospective	AIS	327	Only NL exams	Total spine	1.8%	NR	NR	NR	NR	NR	NR	NR	NR
Davids et al. JBJS-A 2004 [29]	Retrospective	AIS	274	Clonus, abnormal reflexes, muscle weakness, or cavus foot	Total spine	2% entire cohort, 10% of selected patients	NSD	NSD	NSD	NSD	NSD	NSD	NSD	SD
Inoue et al. Spine 2005 [30]	Prospective	All group	250	Hyperactive reflex, ASAR, Muscle atrophy, sensory loss, clonus	Total spine	18%	SD, Early onset < 11 ys	SD, male	SD, left curves	NR	NR	SD- all abnormal neurologic finding	SD	SD, Kyphois >30°
Nakahara et al. Spine 2010 [31]	Retrospective	JIS + AIS	472	Only ASAR	Total spine	3.8%	SD-Early onset < 11 ys	SD-Male	NSD	SD	NR	SD	NR	SD
Rajasekaran et al. Indian J Orthop 2010 [32]	Retrospective ?	All group	94	Muscle weakness, wasting ,ASAR, Babinski	Total spine	16%	SD-Early onset < 11 ys	NR	NSD	NSD	SD	SD	NR	SD

*AIS: Adolescent Idiopathic Scoliosis, JIS: Juvenile Idiopathic Scoliosis, IIS: Infantile Idiopathic Scoliosis. **ASAR: Asymmetric Superficial Abdominal Reflex. †NR: Not Reported in the article. ‡NSD: No Significant Difference. ¥SD: Significant Difference.

Higher incidences were reported in those studies that infantile and juvenile scoliosis were included [8,22,26] as well as those studies in which all patients even with major neurologic signs such as hypereflexia, clonus and muscle weakness, were included [8,30,32]. In this study, only patients with minor neurologic sign (e.g. ASAR) were included, so the incidence of 11.7% of spinal cord abnormalities was reasonable. When the patients who underwent neurosurgical intervention were separated from the others, the incidence decreased to 5 patients (3.5%). All of these patients have had ASAR. The others were not operated for their neuroaxial abnormalities; no neurologic deterioration was detected after their scoliosis surgery.

Regarding the age of the patients, younger age at presentation was considered in most studies as an indicator of neuroaxial abnormalities (Table 3) [30-32]. Although adolescent idiopathic scoliosis was only included in the study, the younger age at presentation was significantly correlated with abnormal neuroaxial abnormalities (*P = 0.05*). It has been concluded that earlier onset scoliosis is associated with a high incidence of neuroaxial abnormalities.

Another important factor is the gender. Male sex has been considered in three studies as a significant factor [27,30,31] but the others did not agree [8,26,29]. Our study could not have revealed the correlation between male gender and abnormal neuroaxial findings.

Atypical curves (curves other than right thoracic major curve) were also studied by many authors but existing studies were reported controversial results (Table 3). Our study was not able to find a significant correlation among various types of curves and direction of the curves with the abnormal neuroaxial findings.

The curve magnitude at presentation was also studied in the literature. But no significant correlation with neuroaxial abnormalities was found in most of them (Table 3) neither in our study [23,26,29,32,33].

There are controversial results according to Davis et al. [29] and Rajasekaran [32] about the double major curves and existing neuroaxial abnormalities. According to our results, more severe lumbar or thoracolumbar curves could increase the probability of neuroaxial abnormalities (*P = 0.03*). Qiao et al. also reported more frequent neural axis abnormalities in thoracolumbar curves [34].

In most studies, persistence of neurologic signs correlates significantly with abnormal MRI findings [30-32]. In our study, we only selected patients with minor neurologic sign (e.g. ASAR), all patients with ASAR has had abnormal MRI findings and most of them were operated by our neurosurgeon.

Absence of thoracic kyphosis is an inherent character of idiopathic curves. Thoracic kyphosis or loss of apical segmental lordosis has been considered recently as an important indicator of an existing neuroaxial abnormality [27,29-32]. Our study was not able to confirm this correlation (*p = 0.2*).

The other indicator which was the focus of our study is the coronal shift. Lee et al. studied the correlation between coronal balance and neuroaxial abnormalities [33], but in their study neurologic examination and presence of ASAR have not been mentioned. They defined coronal imbalance as a coronal shift more than 2 cm and they have not been able to find a significant correlation between neuroaxial abnormalities and coronal imbalance. In our study, the mean of transverse coronal shift from plumb line was 18.5 mm (range 0 to 56 mm) in patients without abnormalities which was significantly lesser than those who had neuroaxial abnormalities with the mean of 37.6 mm (range 34 to 41 mm) (*P = 0.02*). We considered coronal imbalance when lateral shift of the spine was more than 15 mm to increase the sensitivity of selection criteria. Forty-six patients (32%) have had 15 mm or more coronal imbalance. Thirteen of these patients have had neuroaxial abnormalities. Two third of patients with abnormal MRI findings have had coronal imbalance. The severity of coronal imbalance could predict existing neuroaxial abnormalities but neither the severity of neuroaxial problem, nor the necessity of neurosurgical intervention. The significance of the correlation between severe coronal imbalance and/or severe lumbar or TL curves with MRI detected abnormalities, has not been well studied. We think that the discordance between our study and that of Lee et al. is due to the inclusion criteria and the way to define the coronal imbalance.

The headache or backache were not evaluated in our study. It is unusual for an adolescent patient with true idiopathic scoliosis to have significant pain. Scoliosis associated with chronic, disabling pain or pain that awakens the patient at night has been associated with tumors in the spine and spinal cord and warrants further investigation. Morcuende et al. [35] reported 13 scoliosis patients who underwent MRI because of pain. None of them had an abnormal MRI, and none of their patients who had syringomyelia and Arnold-Chiari type I malformations complained of headache or neck pain.

Regarding neurologic examination, only the patients with subtle neurologic findings were included in the study. The others have been referred to the neurosurgery consultation and were not included in the study. There were 6 patients who have had ASAR. All of them have been found to have abnormal neuroaxial finding on their MR imaging. All but one of them were operated by neurosurgeon for extensive syringomyelia or associated Arnold Chiari malformation. Otherwise, eleven patients with abnormal neuroaxial abnormalities (70%) have had normal neurologic examination. This incoherency between abnormal MR findings and physical examination has been reported up to 60% patients with scoliosis secondary to syringomyelia or Chiari malformations [32,36,37]. The clinical significance of syringomyelia or Chiari malformations without any neurologic signs is not fully understood. These patients, who had normal clinical examination with neuroaxial anomaly, underwent arthrodesis without any neurologic complication.

It has been concluded that the most valuable indicator of a surgically important abnormality of the central nervous system is presence of ASAR, and may follow by the presence of a coronal shift. Physical examination findings and subtle clues on diagnostic imaging may help the orthopaedic surgeon diagnose scoliosis associated with syringomyelia or Arnold-Chiari malformation. However, the cause and effect of these neurological abnormalities and scoliosis is not well established. Early age at the onset and structural lumbar or thoracolumbar curves should also consider as a risk factor. The other factors should be studied in a larger series of patients.

## Competing interests

The authors declare that they have no competing interests.

## Authors’ contributions

MK: carried out the study design, gathering data and analyzed them. SS: interpreted all MRIs and X-rays. KM: directed the study, formulated the results and drafted the discussion section. All authors read and approved the final manuscript.
